# Rapid SERS Detection of Thiol-Containing Natural Products in Culturing Complex

**DOI:** 10.1155/2020/9271236

**Published:** 2020-08-01

**Authors:** Yan Hong, Rui Wang, Zhuoran Jiang, Zisong Cong, Heng Song

**Affiliations:** ^1^School of Materials and Energy, University of Electronic Science and Technology of China, Chengdu 610054, China; ^2^College of Chemistry and Molecular Sciences, Wuhan University, Wuhan 430072, China

## Abstract

Thiol-containing natural products possess a wide range of bioactivities. The burst of synthetic biology technology facilitates the discovery of new thiol-containing active ingredients. Herein, we report a sensitive, quick, and robust surface-enhanced Raman scattering technology for specific and multiplex detection of thiol-containing compounds without purification requirements and also indicating the thiols with different chemical environments. Using this platform, we successfully demonstrated the simultaneous detection of thiol-containing compounds from as low as 1 *μ*M of analytes spiked in complex culture matrices.

## 1. Introduction

Over the past several decades, many natural products, such as important antibiotic, anesthetic, antimicrobial, and anticancer compounds, have been isolated from natural microbes [1–4]. Thiols form an important subclass of metabolite molecules in nature [5–9], playing important roles in biological systems associated with cardiovascular diseases [10], Alzheimer's disease [11, 12], leukaemia [13, 14], and cancer [15, 16] to name a few. Thiol-containing pharmaceuticals such as penicillamine, mercaptopurine, and captopril are effective in the treatment of many serious diseases such as arthritis, hypertension, skin disease, and cancer [17, 18]. In addition, ergothioneine, a natural occurring thiol, has been widely employed in health-related products such as antiaging serum, vitamin pills, sun scream, and sports energy drinks [5, 19–21]. Many abovementioned thiol-containing pharmaceuticals or active ingredients are natural products and their analogs. With the development of sequencing technology in recent decades, many new biosynthetic gene clusters from different bacterial species have been sequenced, which provides a huge gold mine for the discovery of new valuable thiol-containing metabolite compounds, or even new drug leads [1, 4, 22–24]. However, traditional separation methods are difficult to identify a number of new potentially bioactive thiol-containing compounds due to their unstability during the extraction process. Besides, traditional separation methods barely provide the information about thiols in different chemical environments, which leads to the problem of duplicated discovery of known thiol-containing compounds.

Hence, rapid and selective methods in detecting thiol-containing compounds, which could also reveal the chemical information about thiols, are extremely desirable. This development could provide rich source to explore the new thiol-containing compounds with biomedical activities. Several techniques have been reported for the analysis of thiols, including high-performance liquid chromatography [25], surface-enhanced Raman spectroscopy (SERS) [26], capillary electrophoresis [27], electrochemical detection [28–30] fluorescence detection [31–35], and mass spectrometry identification [36]. Among these methods, SERS is particularly attractive with the advantages including rapid sampling, predictable fingerprint recognition, and ultratrace detection limit. Especially, thiols are known to interact covalently with noble metal surfaces [37–39], establishing a sulfur-metal bond that has been also exploited for producing self-assembled monolayers on gold nanocavities, which would generally provide escalated SERS signals indicating the information of chemical structure. With natural products involved, multiple implementations have been performed in SERS-based analysis including the study of molecular configurations [40], molecular screening over herbal or plant extracts [41–43], quality control for natural product based medicines [44], and archaeological examination for artificial textiles [45].

Herein, inspired by the sulfur-selective properties of SERS and their special response to thiol analytes, we demonstrated a SERS detection method for thiol compounds that is not only easy to implement with high specificity but also able to differentiate the thiols in different chemical environments.

## 2. Materials and Methods

### 2.1. Synthesis of AuNPs

The AuNPs was synthesized based on the seed-growth method reported by Ziegler and Eychmüller [46]. First, the AuNP seed was prepared in aqueous solution for subsequent growth. 2.5 mL of HAuCl_4_ solution (0.2% w/v) was added to 50 ml of deionized water, were heated to boiling. Then, 2 mL solution containing 0.05% w/v citric acid and 1% w/v sodium citrate was added into the boiling solution under stirring. The solution was kept boiling until the color turned to dark red. The obtained red seed solution was cooled down and sealed for following use.

For seed growing, two types of solution were prepared, including solution A (10 mL of 0.04% w/v HAuCl_4_) and solution B (10 mL containing 0.05% w/v ascorbic acid and 0.025% w/v trisodium citrate).

**Figure 1:**
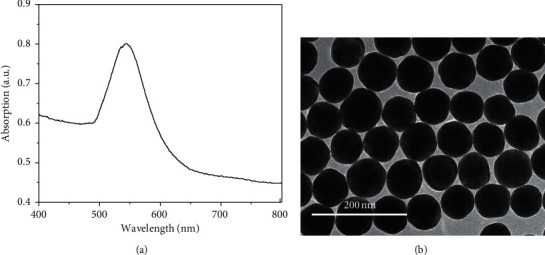
(a) Absorption spectrum and (b) TEM image of synthesized AuNPs.

At the beginning of seed growth, 3 mL of the seed solution was diluted to 20 mL. Next, the prepared 10 mL of solution A and 10 mL of solution B were dropped into the seed solution by Teflon tubes through siphonage. It took about 40 minutes to finish the solution A and B. The mixed solution was then heated to boiling for 20 minutes with stirring. The synthesized AuNPs were characterized by transmission electron microscope (TEM) (Tecnai G2 F20, FEI) and UV-vis spectroscopy (UV-2450, Shimadzu). The obtained colloid contains well-dispersed spherical AuNPs sharing similar radius around 30 nm (Figure 1).

### 2.2. *E. coli* DH5*α* Culture Preparation

*E. coli* DH5*α* strain was streaked on the Luria–Bertani (LB) plate and incubated at 37°C overnight. A single colony was inoculated into a 50 mL LB medium and incubated at 37°C for 18 hours. The supernatant was obtained after centrifugation at 12000 rpm for 20 minutes.

### 2.3. SERS Measurements

The AuNP colloid was condensed from 1 mL into 20 *μ*L by centrifugation (4500 rpm, 5 minutes). 20 *μ*L of target solution (dissolved in DI water or LB supernatant) was mixed with the concentrated AuNPs and dropped onto a cleaned quartz substrate. The sample on the substrate was then dried in a vacuum chamber (0.01 MPa) for 20 minutes. The information of compounds is listed in Table S1.

The SERS measurements were performed by a Horiba spectrometer (iHR 550) and 785 nm diode laser with 120 mW output power. The grating was set with a parameter of 600 gr/mm and slit width of 100 *μ*m. The spectra were taken with the laser spot (20 *μ*m in diameter) focusing on the agglomerates of AuNPs at the perimeter of the droplet through a 100x air objective (NA = 0.68) with the accumulation time of 10 seconds. The total power in the sample plane was 27.5 mW.

## 3. Results and Discussion

### 3.1. Validation and Analysis of SERS Measurements

**Figure 2:**
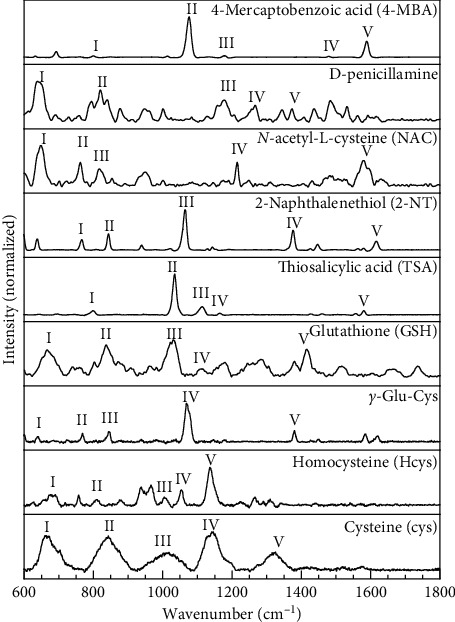
Normalized SERS spectra of all measured chemicals with a concentration of 1.0 × 10^−3^ M. The number marks are corresponding to the Raman peak (I-V) assignments in Table 1.

Initially, to establish the SERS spectral profile of thiol compounds in DI water, we selected 9 thiol-containing compounds, and most of which are typical naturally occurring thiols or analogs, such as cysteine, *γ*-Glu-Cys, glutathione, and homocysteine (Table 1). The aqueous solution with thiol-containing molecules of interest was mixed with the concentrated AuNP colloid. The mixture formed AuNP aggregation after the evaporation of water, simultaneously creating electric field “hot spots” in the vicinities between the AuNPs [60]. The analytes with the thiol group were preferentially absorbed to the surface of metal, and the ones sitting in “hot spots” show highly amplified Raman signal. Those thiol-containing compounds were recognized by SERS, and their Raman spectra were recorded. As shown in Figure 2, all the molecules exhibit characteristic vibration features corresponding to specific molecular bonding (Table 1).

To examine the fingerprint recognition capacity of the SERS response to thiol compounds, we compared the spectra between cysteine and homocysteine, which are only one carbon different with similar chemical structure. The two chemicals share similar Raman features including the strong C-S stretching, NCH bending, and CO stretching band around 670 cm^−1^, 1010 cm^−1^, and 1140 cm^−1^, respectively. The major difference between these two amino acids is spotted at the 1056 cm^−1^ peak of homocysteine for HCH rocking and NCH bending, which is absent in the cysteine spectra. This difference in SERS spectra between two compounds with similar structures could be attributed to varied alignment and molecular dipole of the two chemicals absorbed on the AuNP surface [61, 62]. On the other hand, the signal broadening at higher concentration was observed in the spectra of the analytes, such as GSH and Cys (Figure 2, S1). This phenomenon is the result of more averaged molecule orientation and more complex local chemical environment in the samples of higher concentration [63]. In particular, alkyl thiols and aromatic thiols exhibit different SERS response where aromatic thiols provide higher signal intensity and better signal-noise ratio.

As shown in Figure 2, the compounds with the thiophenol group including thiosalicylic acid (TSA), 2-naphtalenethiol (2-NT), and 4-mercaptobenzoic acid (4-MBA) demonstrate superior signal-to-noise ratio compared with the others. One of the reasons is that there are multiple binding sites, such as benzene-*π* system and carboxyl and thiol groups for the adsorption of these molecules on metal surface [51, 64]. The AuNP surface with escalated electric field intensity provides electromagnetic enhancement of Raman scattering for the molecules [65].

The sensitivity of the conducted SERS method was calculated as the minimum amount of thiol-containing amino acid required to achieve recognizable peak with the signal-to-noise ratio higher than 3. Among 9 thiol-containing molecules tested, the determined detection limits vary from 1 *μ*M to 1 pM, with one lowest detection limit as low as 1 fM for 2-NT, which further confirms the better SERS response from aromatic thiols (Table 2). It is important to note that the SERS method presents a generally weaker response towards nonthiol biological molecules other than thiols with higher detection limits or signals [66].

Because of the special confinement of enhanced near field volume, the amplified inelastic scattering intensity is only from the molecules close to the surface of metal nanoparticles within a few of nanometers [51]. Therefore, the spectral features of mixture of multiple ingredients are usually dominated by particular compounds that strongly absorb to the metal nanoparticle surface. Notably, the SERS method is able to differentiate the fingerprint feature of thiol molecules without the interference from other thiol compounds, which is the foundation for the SERS method to identify the thiols using their characteristic spectral responses. In order to demonstrate the potential application of the SERS detection method in more complicated situation, three pairs of compounds from the 3 subject molecules (TSA, 4-MBA, and 2-NT) with an aromatic thiol group were mixed and measured.

**Figure 3:**
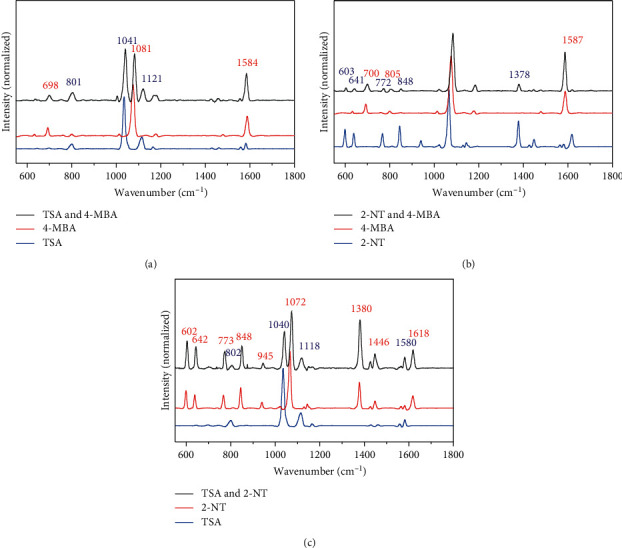
SERS spectra of the solutions containing two chemicals and corresponding spectra of individual chemicals. (a) TSA (1.0 × 10^−4^ M) and 4-MBA (1.0 × 10^−5^ M); (b) 2-NT (1.0 × 10^−5^ M) and 4-MBA (1.0 × 10^−4^ M); (c) TSA (1.0 × 10^−4^ M) and 2-NT (1.0 × 10^−5^ M).

For each pair, the resulting SERS spectra reserved distinctive signal peaks of both molecules (Figure 3). The absence of spectral interference is due to their similar molecular structures, which contributes to the similar binding affinities to AuNPs. As a result, this strategy quickly eliminates the compounds which are already discovered in thiol-containing pharmaceutical biosynthesis or fermentation, providing a threshold to determine the candidates for further purification and detailed characterization. Therefore, this approach is able to avoid the problem of duplicated discovery of known thiol-containing compounds.

**Figure 4:**
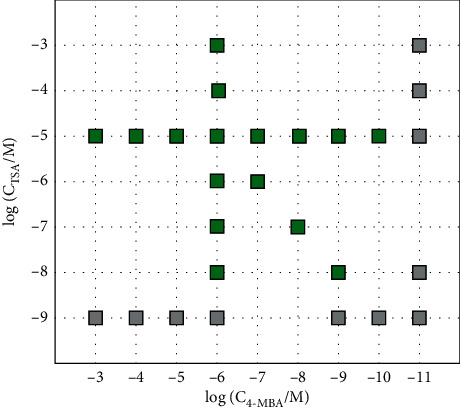
Detection limit map showing different combination of TSA and 4-MBA concentrations. The squares are marks showing the detection results: green, both detectable in spectra; grey, fail to recognize both of the analytes.

However, because of the varied binding affinities of the subject to the AuNP surface, only the mixed solution within particular proportional combination yields recognizable spectral features of both analytes.  Figure 4 illustrates the range of concentration combinations for TSA and 4-MBA which can be both recognizable by the SERS measurement. It is found that for concentrations of TSA > 10^−8^ M and 4-MBA > 10^−10^ M in the mixed solution, the major spectral feature of both components was detected, indicating the coexistence of the two kinds of molecules. Although this detection limit for mixed solution is substantially higher compared with that in monotonic solution as shown in Table 2, the detection limit values are sufficient for the laboratory assessment in detection of thiol-containing natural products or fermentation production of thiol-containing compounds.

### 3.2. Detection of Target Molecules in Culture Medium

**Figure 5:**
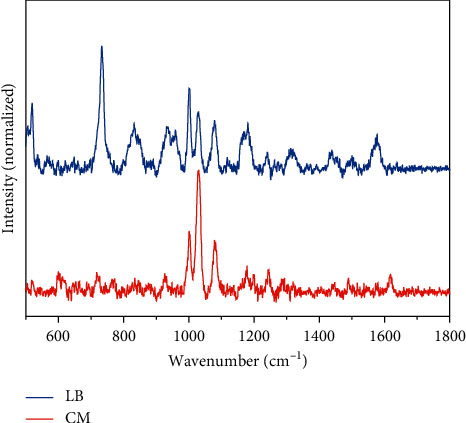
SERS spectra of the initial LB medium (LB) and medium after bacteria culturing (CM).

For rapid detection of thiol-containing natural products, it is normally needed to identify the compounds from a culture medium, which contains a variety of amino acids, sugars, salts and peptides, etc. Without specific extraction, some molecules may exhibit stronger SERS response that covers the spectral feature of target molecules. Also, the physical absorption of other molecules from background may block the “hot spot” near the AuNP surface, weakening the Raman scattering intensity of analytes. Therefore, an investigation to evaluate interference from a culture medium was carried out. To examine whether the secretion of metabolites generated from strains affects the SERS-based detection, *E. coli* DH5*ɑ* cultures were grown under standard conditions and the supernatant of the cultured medium were obtained. As shown in Figure 5, the LB medium spectra differ drastically before and after the culturing of bacteria. The reduced number of Raman features could be due to the decomposition of the nutrients in the LB medium. The metabolic activity of the bacteria yielded generally small carbohydrate waste which is often Raman insensitive [67].

**Figure 6:**
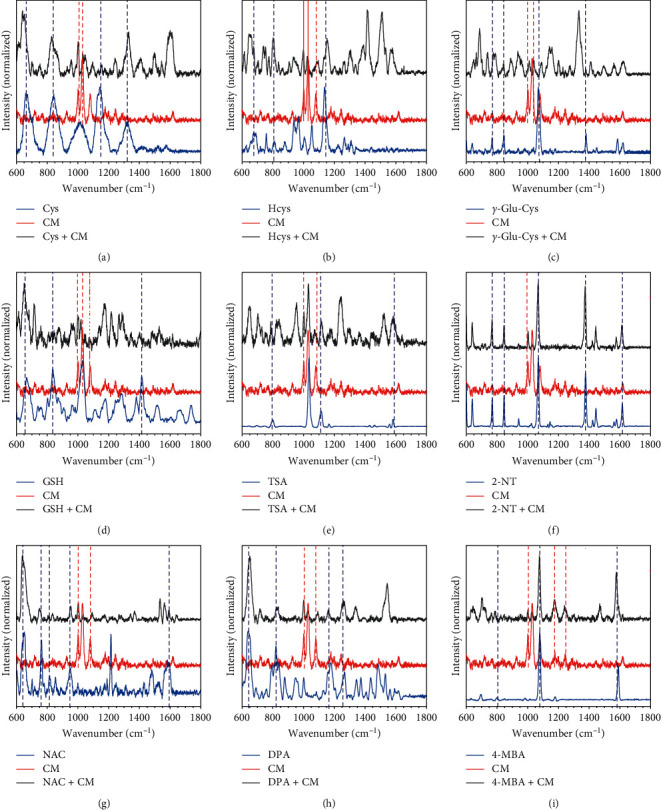
(a–i) SERS spectra of target thiols in the bacterial medium (black) in comparison with the SERS spectra of pure thiol (blue) and the cultured bacterial medium (CM, red). The concentrations of the target thiols in CM are 5.0 × 10^−6^ M for (a, b, d, e, f, and i) and 5.0 × 10^−5^ M for (c, g, and h). The dotted lines mark the corresponding SERS features.

When the target compounds were spiked to the cultured medium, the SERS-based method was able to provide its expected Raman spectra with a detection limit as low as the magnitude of 10^−6^ M. As illustrated in Figure 6, the characteristic features of the target molecules are mostly recognizable in the spectra of mixed samples. Although the bands around 1000–1100 cm^−1^ could interfere with particular peaks because of the signals from culture medium, the other recognizable bands beyond this range are still helpful in identifying the targets. For fermentation production processes, the concentration of the target compounds should be at least 1 mg/L or higher, which is worthwhile for the following optimization process. Considering the average molecular weight of target compounds, 1 mg/L corresponds to the scale of 10^−5^ M, which is higher than the detection limit of our method from the sample with the culture medium. Therefore, this observation demonstrates that this SERS-based method is applicable for direct detection of thiol-containing compounds in complex matrices as the cultured medium. In summary, the devised strategy can readily enable discovery of thiol-containing compounds from complex culture matrices.

## 4. Conclusions

In this work, we developed a rapid and effective SERS-based method for identifying thiol-containing natural products. Using this method, we successfully detected and differentiated thiol-containing compounds. The limit of detection of our SERS-based method in the fermented medium is at the level of 10^−6^ M. Taken together, this system provides an easy and fast methodology for detecting thiol-containing compounds, which represents a promising new method to assist for exploring the chemical space of thiol-containing compounds with biomedical activities.

## Figures and Tables

**Table 1:** The SERS peak assignments of chemicals listed in Figure 2.

		I		II		III		IV		V	
Chemical	Molecular structure	*R*_S_ (cm^−1^)	Assignment	*R*_S_ (cm^−1^)	Assignment	*R*_S_ (cm^−1^)	Chemical	*R*_S_ (cm^−1^)	Assignment	*R*_S_ (cm^−1^)	Assignment
Cysteine (Cys) [47–49]	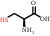	665	*ν*(C-S)	837	*δ*(HCS)	1014	*δ*(NCH) + *δ*(HCH)	1156	*ν*(C-O) + *δ*(NCH)	1325	*δ*(NCH) + *δ*(HCH)

Homocysteine (Hcys) [47–49]	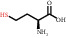	676	*ν*(C-S)	818	*δ*(HCS)	1004	*δ*(NCH) + *δ*(HCH)	1056	*δ*(NCH) + *δ*(HCH)	1137	*ν*(C-O) + *δ*(NCH)

*γ*-Glu-Cys [47, 49]	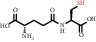	640	*ν*(C-S)	767	*δ*(C-SH)	842		1068	*δ*(NCH) + *δ*(HCH)	1380	

Glutathione (GSH) [50]	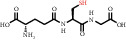	663	*ν*(C-S)	835	*ν*(C-CN)	1022	*ν*(C-N)	1115	*ν*(N-C) + *ν*(C-C)	1414	*ν*(OCO)

Thiosalicylic acid (TSA) [51–53]	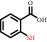	797	*ν*(C-COOH)	1035	Ring breath	1114		1161	*δ*ip(C-H)	1578	Ring stretch

2-Naphthalenethiol (2-NT) [54]	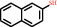	768	*δ*_wag_(C − H)	845	*δ*_twist_(C − H) (C-H)	1064	*δ*(C-H)	1377	Ring stretch	1619	Ring stretch

*N*-acetyl-L-cysteine (NAC) [47, 55]	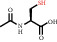	652	*ν*(C-S)	763	*δ*(C-SH)	818	*δ*(HCS)	950	*δ*(NCH) + *δ*(HCH)	1582	Amide I

D-penicillamine (DPA) [56]	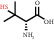	638	*ν*(C-S)	819	*δ*ip(C-H)	1182	*δ*ip(C-H) + *ν*(C-N)	1267	*δ*(C-H)	1374	*ν*(OCO)

4-Mercaptobenzoic acid (4-MBA) [57–59]	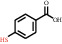	797	*δ*(OCO)	1074	Ring stretch	1179	*δ*(C-H)	1479	*ν*(C-C) + *ν*(C-H)	1588	Ring stretch

Abbreviations: *R*_s_, Raman shift; *ν*, stretching; *δ*, bending; ip, in plane.

**Table 2:** Detection limit of target chemicals in DI water with SERS analysis.

Chemical name	Detection limit (mol/L)
Cysteine (Cys)	1.0 × 10^−8^
Homocysteine (Hcys)	1.0 × 10^−8^
*γ*-Glu-Cys	1.0 × 10^−6^
Glutathione (GSH)	1.0 × 10^−6^
Thiosalicylic acid (TSA)	1.0 × 10^−12^
2-Naphthalenethiol (2-NT)	1.0 × 10^−15^
*N*-acetyl-L-cysteine (NAC)	1.0 × 10^−10^
D-penicillamine (DPA)	1.0 × 10^−9^
4-Mercaptobenzoic acid (4-MBA)	1.0 × 10^−12^

## Data Availability

The data used to support the findings of this study are available from the corresponding author upon request.

## Supplementary Materials

Supplementary Materials Supplementary Figure S1: (left, a1–i1) SERS spectra of compounds in Table 1 with varied concentration; (right, a2–i2) average peak intensities as a function of concentration for corresponding compounds. Table S1: information of compounds.
