# Supplementary material for: Rapid SERS Detection of Thiol-Containing Natural Products in Culturing Complex
Source: Int J Anal Chem. 2020 Aug 1;2020:9271236. doi: 10.1155/2020/9271236 (PMC7416272; doi:10.1155/2020/9271236)
Supplement: Supplementary Materials — Supplementary Figure S1: (left, a1–i1) SERS spectra of compounds in Table 1 with varied concentration; (right, a2–i2) average peak intensities as a function of concentration for corresponding compounds. Table S1: information of compounds. [file 9271236.f1.docx]

**Supporting Information**

**Rapid SERS Detection of Thiol-Containing Natural Products in Culturing Complex**

Yan Hong^1^, Rui Wang^1^, Zhuoran Jiang2, Zisong Cong2 and Heng Song ^2^,

1 School of Materials and Energy, University of Electronic Science and Technology of China, 610054, Chengdu, China.

2 College of Chemistry and Molecular Sciences, Wuhan University, 430072,Wuhan,China.

E-mail: hengsong@whu.edu.cn

Table S1. Information of compounds.

| **Compound** | **Supplier** | **Purity** |
| --- | --- | --- |
| Cysteine | Shanghai Aladdin Biochemical Technology Co., Ltd. | 99% |
| Homocysteine |  | [95.0%](https://www.aladdin-e.com/zh_cn/l134501.html) |
| γ-glu-cys |  | 80% |
| Glutathione |  | 98% |
| Thiosalicylic acid |  | 99% |
| 2-Naphthalenethiol |  | 99% |
| N-Acetyl-L-cysteine |  | 99% |
| D-Penicillamine |  | 98% |
| 4-Mercaptobenzoic acid | Energy Chemical Co., Ltd. | 90% |
| Citric acid | ChengDu Chron Chemicals Co,.Ltd | 99.8% |
| Trisodium-citrate |  | 99% |
| Ascorbic acid |  | 99.99% |
| Tetra chloroauric acid trihydrate (HAuCl_4_·3H_2_O) | Adamas Reagent Co.,Ltd. | 99.9% |


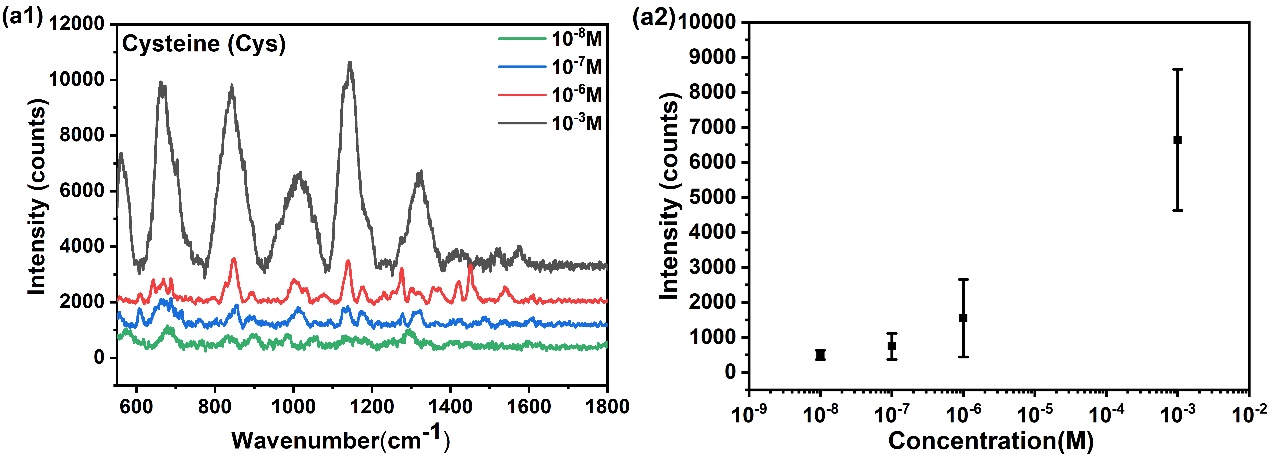


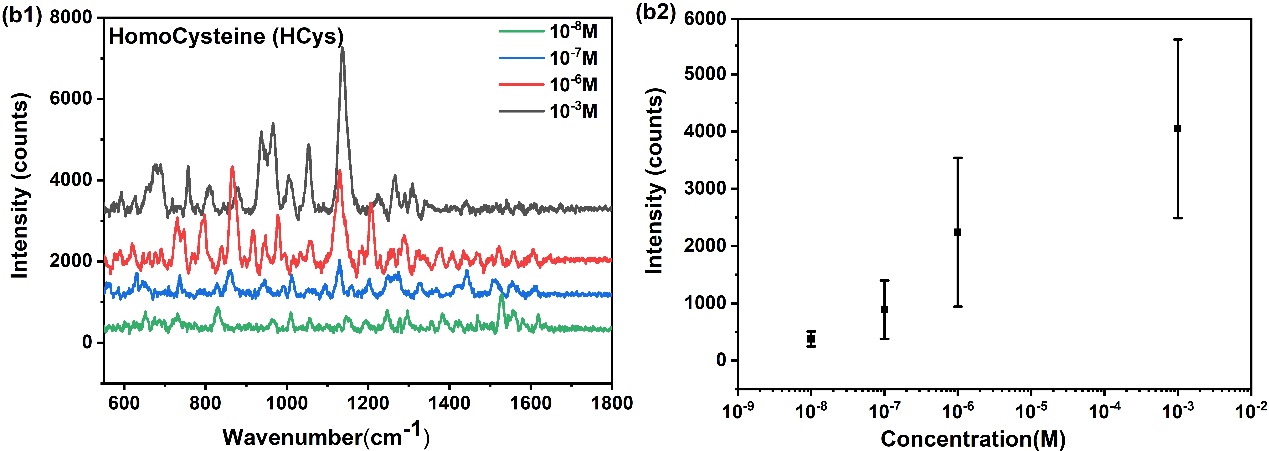


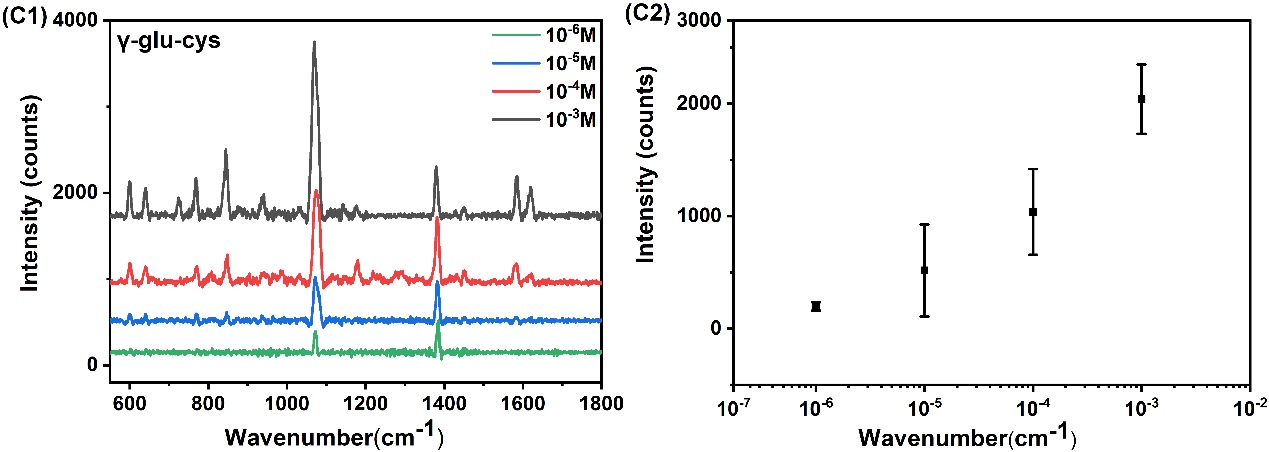


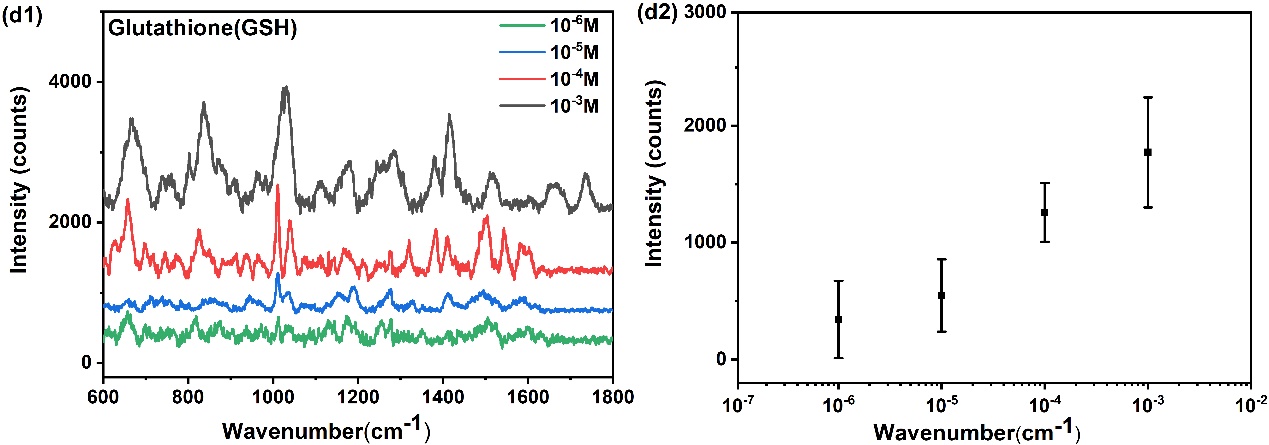


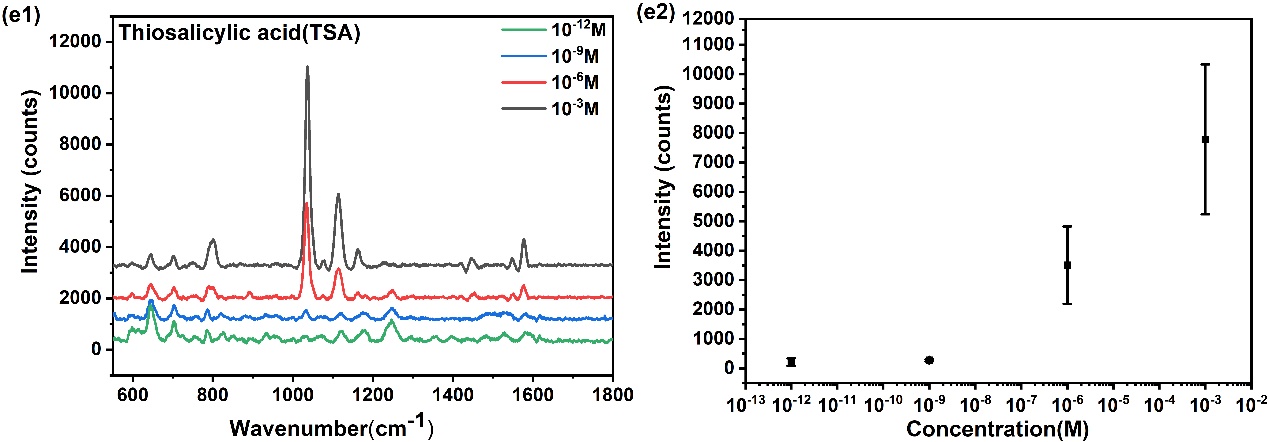


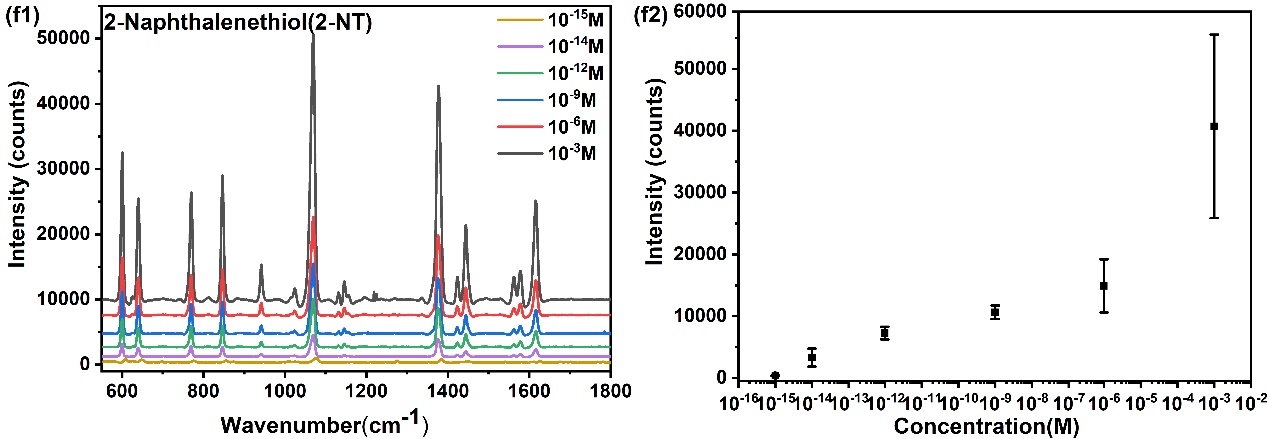


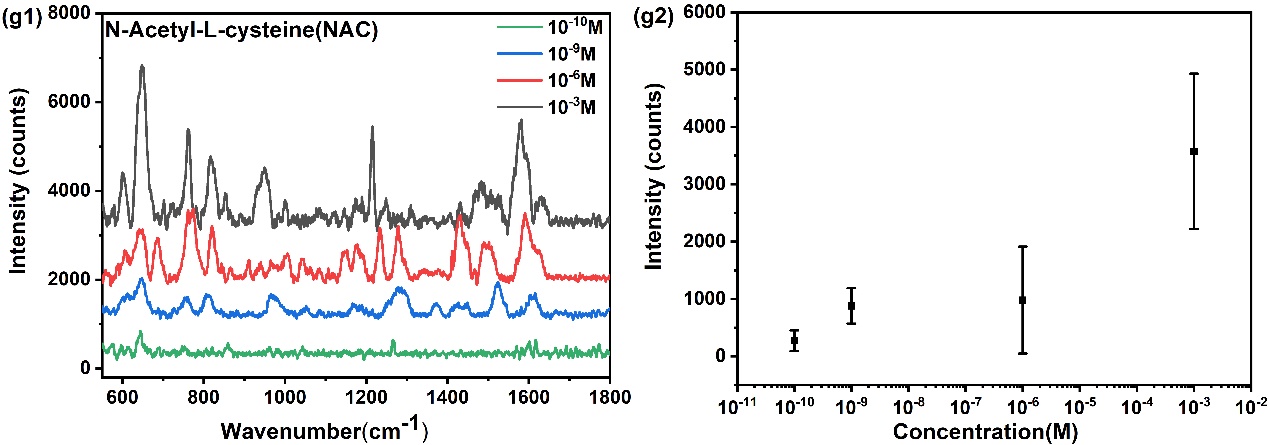


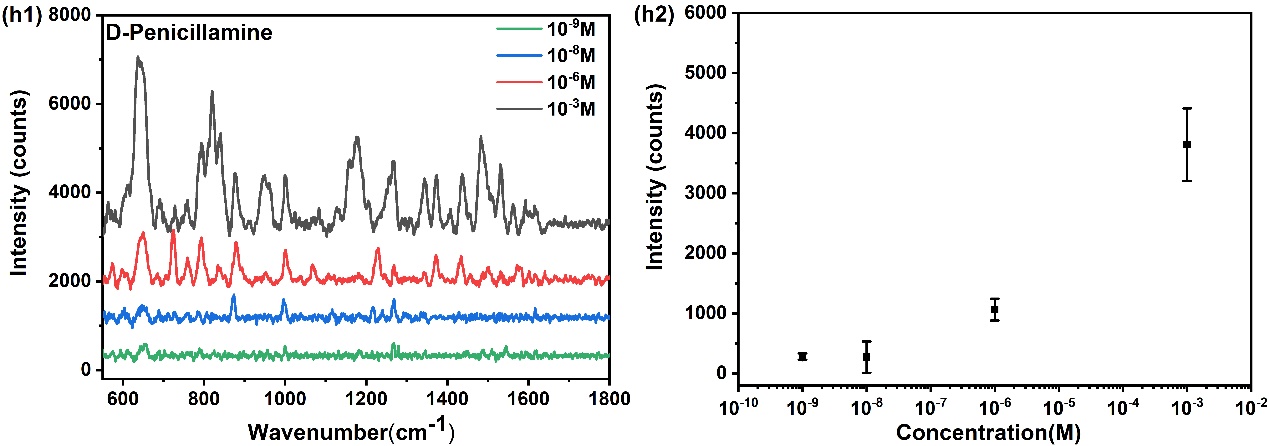


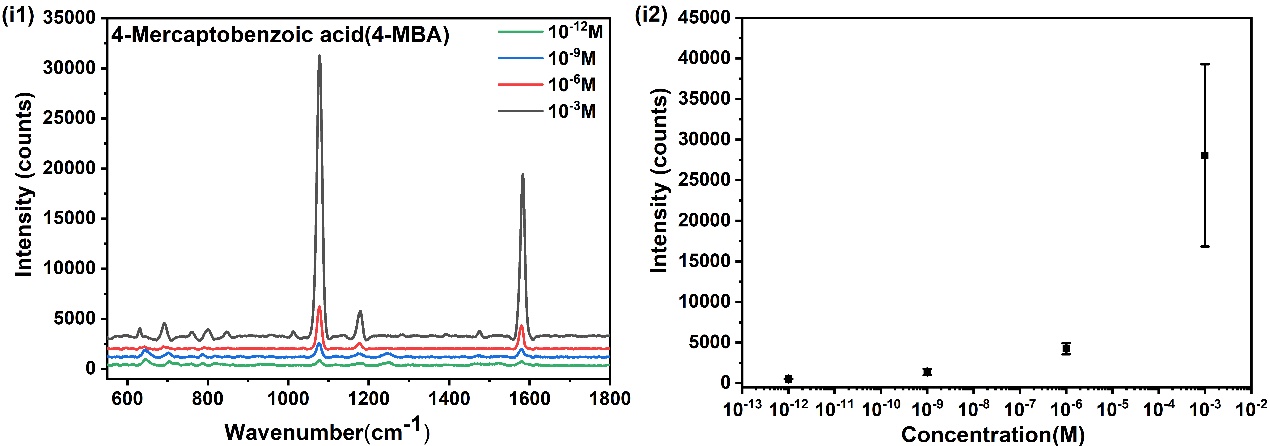


Figure S1. Spectra of analytes with varied concentration. (Left, a1-i1) SERS spectra of different compounds in Table 1 with varied concentration. (Right, a2-i2) Average peak intensities as a function of concentration for corresponding compounds. The selected peaks are: 837 cm^-1^ for cysteine, 1137 cm^-1^ for homocysteine, 1068 cm^-1^ for γ-glu-cys, 1022 cm^-1^ for glutathione, 1035 cm^-1^ for thiosalicylic acid, 1064 cm^-1^ for 2-Naphthalenethiol, 652 cm^-1^ for N-Acetyl-L-cysteine, 655 cm^-1^ for D-penicillamine and 1074 cm^-1^ for 4-Mercaptobenzoic acid, respectively.
